# Near Azeotropic Ethanol–Water Mixture Pervaporation Through a Polyvinyl Alcohol Membrane: A Parametric Study on Process Efficiency

**DOI:** 10.3390/polym18010065

**Published:** 2025-12-25

**Authors:** Cristiana Luminița Gîjiu, Daniel Dumitru Dinculescu, Raluca Isopescu

**Affiliations:** Faculty of Chemical Engineering and Biotechnologies, National University of Science and Technology POLITEHNICA Bucharest, 011061 Bucharest, Romania; luminita.gijiu@upb.ro (C.L.G.); raluca.isopescu@upb.ro (R.I.)

**Keywords:** PVA membrane, COMSOL modeling and simulation of ethanol–water pervaporation, multiobjective optimization of purity and productivity

## Abstract

The goal of this study was to explore how different operating parameters influence the performance of a polyvinyl alcohol (PVA) membrane in pervaporation for separating ethanol–water mixtures. Specifically, the focus was on understanding how variations in feed composition, temperature, and permeate pressure affect the separation efficiency. The study aimed to provide a range of operating conditions that offer a balance between maximizing both the purity and quantity of ethanol. This was achieved through statistical models, which were generated by simulating the pervaporation process under various conditions using COMSOL Multiphysics^®^ 6.3 and following a Box–Behnken design. It was found that similar operating conditions (temperature ~100 °C; pressure ~4–5 kPa) are suitable for both kinds of mixtures near azeotrope, with higher water content (~0.15 mass fraction) and lower water content (~0.05 mass fraction) obtaining very high recuperation degrees (generally above 99%). For more concentrated solutions (lower water content), it was possible to obtain optimal trade-off solutions (separation degree vs. retentate enrichment in ethanol), even at lower temperatures (~80 °C).

## 1. Introduction

There is a wide variety of processes that lead to the obtaining of azeotropic mixtures. Perhaps the best-known case is the ethyl alcohol/water azeotropic mixture, which needs to be separated in cases where (bio)ethanol is intended to be used as a (bio)fuel, for example. The butanol/water azeotrope also belongs to the same category [1]. As expected, the chemical industry also abounds in examples of azeotropic mixtures that appear in various manufacturing processes of certain chemical products: dimethyl carbonate/methanol [2,3], methanol/methyl-tert-butyl-ether [4], ethanol/ethyl-tert-butyl-ether [5,6], benzene/cyclohexane [7], etc.

The separation processes of azeotropic mixtures are usually energy and/or material-intensive (as is the case of the solvent used in separation by liquid–liquid extraction [8,9], extractive distillation [9,10,11,12], or azeotropic distillation [9,12]). The separation processes are usually performed in two steps: a first step involving separation by classical processes until concentrations close to the azeotrope concentration are obtained, followed by a second step involving breaking the azeotrope and purifying the components. The first way to approach the separations of liquid mixtures (of course, where the difference between the boiling points of the components allows it) was (and still is) distillation/rectification [8,9,10,11,12].

However, this separation option is only useful until the concentration corresponding to the azeotropic mixture is obtained. Therefore, to obtain pure components from such a mixture, membrane processes, in particular pervaporation, have been proposed [13,14]. Pervaporation does not suffer from the thermodynamic limitations that occur in the case of azeotropic mixtures, generally relying on the solubilization–diffusion mechanism of the mixture compounds in the membrane material. Hence, there is a need to obtain (and to obtain at the industrial level, not in the laboratory) membranes with high selectivity (high product quality) and ensure high permeate flows (high productivity). However, designing modules for optimal operation is also essential in convincing the industry to modernize [15,16].

A recent study [17] presents the evolution in the period 2002–2021 of the number of publications (articles, reviews, patents, books, conferences, etc.) relating to pervaporation membranes compared to pervaporation modules, i.e., the equipment that actually works to achieve separations by pervaporation (tubular, spiral, hollow-fiber). In a year, if the research to obtain membrane materials is in the order of hundreds, those for the design and study of the functioning of separation modules are barely in the order of tens. A possible explanation could be the reluctance of the industrial environment to replace classic equipment with modern separation equipment [14], apparently not yet convinced of its performance. However, the same authors present a comparative energy consumption for the dehydration of 100 kg of isopropanol starting from the azeotrope containing 88% isopropanol by mass. While in the case of azeotropic distillation and adsorption, it is 36 kWh and 29 kWh, respectively, in the case of pervaporation, it is only 12 kWh.

Recent European regulations require that economic, environmental, and sustainability criteria should be considered when selecting the most appropriate separation methods. Therefore, it has become imperative to replace outdated techniques in certain separation processes with modern techniques that comply with these new regulations.

Over the past two decades, extensive theoretical and numerical studies have been devoted to the modeling of pervaporation processes, with rigorous solutions of the governing transport equations implemented using analytical approaches and computational platforms, such as MATLAB, COMSOL, and CFD-based solvers [18,19,20,21,22,23,24,25,26,27,28]. In this context, most studies emphasize on the description of module hydrodynamics and focus on mass transport mechanisms (e.g., the solution–diffusion model) and their influence on membrane separation properties (selectivity and permeability) [29,30,31,32,33,34,35,36,37,38,39]. However, there has been limited attention to the integrated modeling of heat transfer during the pervaporation process [27,28,34,35,36,37,38]. Furthermore, these studies typically do not incorporate heat transfer along the entire pervaporation module but focus only on the membrane itself [38]. This is surprising given the negligible thickness of the selective membrane layers relative to the module length and the importance of heat for vapor formation on the permeate side. Efficient heat transfer management can significantly impact the overall energy efficiency of the process. By neglecting heat transfer, the process dynamics are only partially understood. The lack of focus on thermal coupling in pervaporation models represents a notable gap in the literature, especially considering the growing interest in energy-efficient membrane systems for large-scale industrial applications. The only comprehensive approach identified in the literature, which integrates flow, mass, and heat transfer for more accurate system performance predictions, is found in [27,28,34,35,36,37], and none of these refer to a PVA membrane. This approach offers valuable insights for optimizing pervaporation processes, aiming for energy reduction and improved productivity.

Regarding the membranes studied in pervaporation processes, they are different, but the vast majority are polymeric: hydrophilic (polyvinyl alcohol (PVA), polyelectrolyte complexes (PECs), chitosan (CS), cellulose derivatives, polyamide (PA), polyimide (PI), etc.) or hydrophobic (poly(dimethylsiloxane) (PDMS), poly[1-(trimethylsilyl)-1-propyne] (PTMSP), polyvinyl chloride (PVC), etc.) [40,41,42].

PVA is widely regarded as the polymer of choice for pervaporation membranes because it combines a set of material properties that match the requirements for efficient separation of water–organic mixtures. By itself, it is not used as a standalone pervaporation membrane because it swells significantly in water, weakening its mechanical properties and reducing its effectiveness [43]. To address this, PVA is typically modified or crosslinked to improve stability and performance [44,45]. The pronounced hydrophilicity of poly(vinyl alcohol)–based membranes confers a strong thermodynamic affinity for water, resulting in high water sorption and permeability and, consequently, preferential water transport under pervaporation conditions [46,47,48,49,50,51,52]. This intrinsic water selectivity underpins their widespread application in the dehydration of alcohols and other organic mixtures, as well as in emerging pervaporation-driven desalination processes, where transport performance is strongly modulated by membrane modification strategies and operating conditions [53,54,55,56,57]. Moreover, PVA has excellent film-forming ability and mechanical stability, allowing the fabrication of dense, defect-free composite membranes (flat-sheet, tubular, or hollow-fiber) with controlled thickness and uniformity [47,49,50]. Its chemical resistance and ability to be chemically modified or crosslinked further enhance its durability, reduce excessive swelling, and improve long-term performance under pervaporation conditions [48,49,55]. Additionally, as reported in the recent review on pervaporation–desalination membranes [53], PVA remains “the main polymer used to make high flux pervaporation membranes,” underscoring its prevalence and general acceptance in both research and industrial applications.

Unsurprisingly, according to what was presented above, PVA was the polymer for the very first commercial pervaporation membranes. In the early 1980s, Gesellschaft für Trenntechnik (GFT) developed a composite membrane featuring a thin, crosslinked PVA selective layer supported on a porous poly(acrylonitrile) substrate. This membrane, deployed for alcohol–water dehydration (e.g., ethanol), marked the transition of PV from laboratory studies to industrial-scale use [41,57]. Since then, PVA-based membranes (including commercially available products, such as the PERVAP™ line [52,56,57]) have remained the benchmark hydrophilic membranes for solvent dehydration and other pervaporation applications [41].

Response Surface Methodology (RSM) offers a robust approach for modeling the relationships between multiple factors and responses, enabling the identification of optimal operating conditions for enhanced process performance [58,59]. In the context of pervaporation, RSM has been applied to optimize membrane materials [60,61] and process variables and system performance [62,63,64,65], particularly for organic solvents and alcohol–water mixtures.

## 2. Models and Solving Methods

### 2.1. Pervaporation Theoretical Model in COMSOL Multiphysics^®^

The separation process was quantitatively described using the flow, heat, and mass transfer equations, particularized for a PVA membrane as a very thin layer on a porous support. According to several research studies [47,49,50], the support reduces the degree of swelling. However, specific values have not been mentioned. The membrane permeance used in the present study was estimated by [57] from experiments reported in [52] for a commercial PVA membrane. The expression of permeance is a function of water activity (a_w_) and temperature (T), and, consequently, it also encompasses the possible swelling (Table 1).

#### 2.1.1. Geometry

**Figure 1:**
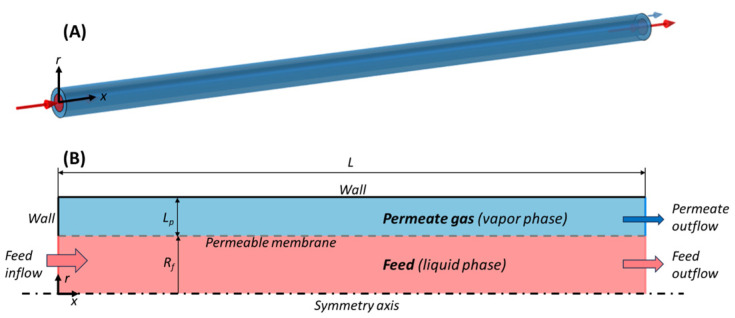
Schematic representation of membrane module geometry. (**A**) Three-dimensional tube-in-tube configuration; (**B**) two-dimensional axisymmetric model representation with the assigned domains, boundaries, and dimensions. The cylindrical geometry spans along directions *x* and *r*.

The pervaporation module consists of a cylindrical tube-in-tube configuration of length L with an active separation layer of PVA. The liquid feed (ethanol with a small fraction of water) flows in the lumen, with radius R_f_, while the gaseous permeate (mostly water) is collected in the outer shell of the tubular pervaporation device, with thickness L_p_. Therefore, the model is built on an axisymmetric geometry, with radial direction r and axial direction x (Figure 1).

#### 2.1.2. Fluid Dynamics

*Feed side.* The feed is a liquid mixture of ethanol and water that flows within the lumen of the membrane module. At the operating conditions (Reynolds number Re_f_ = 474, flow velocity 2 cm/s), the flow is laminar, incompressible, and stationary, and is thus represented by the Navier–Stokes equations of momentum conservation (1) and continuity (2) as follows:

(1) $$\rho_{f}u_{f}\cdot \nabla u_{f}=-\nabla p_{f}+\nabla \cdot \left(\eta_{f}\left(\nabla u_{f}+\nabla u_{f}^{T}\right)\right)$$

(2) $$\nabla \cdot u_{f}=0$$

where $u_{f}=\left(u_{f,r},u_{f,x}\right)$ is the vector of fluid velocity and p_f_ is the pressure in the feed domain *f*. The physical properties, density ρ_f_, and viscosity η_f_, are calculated functions of the local mixture composition and temperature (Table 1).

The flow is driven by setting a fully developed laminar flow with an average velocity in the inlet (x = 0) and an outlet (x = L), with gauge pressure set to a reference value of zero, while axial symmetry applies at r = 0. On the permeable wall (r = R_f_), the total volumetric flux of water and ethanol is a velocity imposed on the radial component of the feed velocity, $\left(u_{f,r},\;\;u_{f,z}\right)=\left(J_{f,v},\;\;0\right)$ i.e., normal to the boundary. The volumetric flux of liquid permeate, $J_{f,v}=J_{w}/\rho_{w,L}+J_{e}/\rho_{e,L}$, results from the mass fluxes of the two components, $J_{w}=N_{w}\cdot \;M_{w}$ and $J_{e}=N_{e}\cdot \;M_{e}$. The molar fluxes N_w_ and N_e_ are expressed as a function of membrane layer permeabilities for water and ethanol, P_m,w_ and P_m,e_, depending on mass fraction of water in liquid w_f,w_ (Table 1), membrane active layer thickness L_m_, and driving force of permeation that is the difference in partial pressures between feed and permeate [66].

(3) $$N_{w}=\frac{P_{m,w}}{L_{m}}\left(\gamma_{w}x_{f,w}P_{v,w}-x_{p,w}p_{p}\right)$$

(4) $$N_{e}=\frac{P_{m,e}}{L_{m}}\left(\gamma_{e}x_{f,e}P_{v,e}-x_{p,e}p_{p}\right)$$

For water (wb) and ethanol (e), γ in Equations (3) and (4) is the activity coefficient function of mole fractions in the liquid feed, x_f_, and P_v_ are the vapor pressures functions of the feed temperature T_f_, x_p_ is the mole fraction in the vapor (permeate) phase, and p_p_ is the total permeate pressure. The expressions of activity coefficients (NRTL relations) and vapor pressures (Antoine-like equations), as well as the other parameters, are listed in Table 1.

*Permeate side.* The permeate is a gaseous mixture of ethanol and water vapors that flows within the space created between the concentric tubes. The laminar gas flow is also stationary, but compressible, and is thus represented by momentum and mass conservation in Equations (5) and (6) as follows:

(5) $$\rho_{p}u_{p}\cdot \nabla u_{p}=-\nabla p_{p}+\nabla \cdot \left(\eta_{p}\left(\nabla u_{p}+\nabla u_{p}^{T}\right)-\frac{2}{3}\eta_{p}\left(\nabla \cdot u_{p}\right)I\right)$$

(6) $$\nabla \cdot \left(\rho u_{p}\right)=0$$

where $u_{p}=\left(u_{p,\;\;r},\;\;u_{p,\;\;x}\right)$ is the vector of fluid velocity and p_p_ is the pressure in the permeate domain. The gas density ρ_p_ is calculated according to the ideal gas law, and viscosity η_p_ results from a mixing law. Both function as the local mixture composition and temperature (Table 1).

The gas outlet (x = L) is defined by imposing a very low (close to vacuum) absolute pressure, p_p_ = p_p,out_. The opposite wall (x = 0) and the lateral wall (r = R_f_ + L_p_) are sealed, with zero gas velocity. On the permeable surface (r = R_f_), the same total volumetric flux of water and ethanol coming from the feed is imposed on the radial permeate velocity, $\left(u_{p,r},u_{p,z}\right)=\left(J_{f,v},0\right)$.

#### 2.1.3. Mass Transfer

*Feed side.* The mass balance for ethanol is set as a function of mass fractions w_f,e_ and w_f,w_ given that the mixture cannot be treated as a very diluted solution of water in ethanol. The convective transport is supported by the liquid flow velocity u_f_, while diffusion was considered according to a Fick’s law approximation and was driven by the mass fraction gradient and was proportional to the water–ethanol diffusion coefficient D_f,we_ as follows:

(7) $$-\nabla \cdot \left(D_{f,we}\nabla w_{f,e}\right)+u_{f}\cdot \nabla w_{f,e}=0$$

This simplified form of mass transport in binary mixtures, represented by Equation (7), produced the same results as the rigorous but much more complicated Maxwell–Stefan approach in our model tests, and no mixture diffusion correction flux was needed. With the computed distribution of w_f,e_, the remaining mass fraction of water results from the mass constraint w_f,w_ =1 − w_f,e_. The mass fractions and mole fractions are interconvertible with $x_{f,e}=\frac{w_{f,e}}{M_{e}}M_{f}$ and x_f,w_ =1 − x_f,e_, where the average molecular weight of the mixture is $M_{f}={\left(\frac{w_{f,e}}{M_{e}}+\frac{w_{f,w}}{M_{w}}\right)}^{-1}$.

The mass fraction in the liquid inlet (x = 0) is w_f,e_ = w_f,e,in_, supplied from a specified mole fraction x_f,e_ = x_f,e,in_. In the outflow (x = L), the normal diffusion flux is zero (i.e., only convection is allowed, and the normal mass fraction gradient is null, $\nabla w_{f,e}=0$). The usual axial symmetry applies in the center of the feed pipe (r = 0). The mass fluxes of ethanol and water at the permeable boundary (r = R_f_) are set to equal the diffusive fluxes corrected with the Stefan velocity (the total mass flux exiting through the membrane divided by density) as follows:

(8) $$\rho_{f}D_{f,we}\nabla w_{f,e}+\left(J_{w}+J_{e}\right)\;w_{f,e}=J_{e}$$

(9) $$\nabla \cdot \left(\rho u_{p}\right)=0$$

The liquid density was computed for the mixture, dependent on local values of liquid composition and temperature (Table 1).

*Permeate side.* The mass balance for ethanol is set similarly to that in the liquid side, and functions of mass fractions in the permeate are w_p,e_ and w_p,w_. The convective transport is driven by the vapor velocity **u**_p_, and the diffusion flux was again approximated by Fick’s law from the mass fraction gradient and the water–ethanol diffusion coefficient D_p,we_ in the vapor phase.

(10) $$-\nabla \cdot \left(\rho_{p}D_{p,we}\nabla w_{p,e}\right)+\rho_{p}u_{p}\cdot \nabla w_{p,e}=0$$

No sensible differences appeared when using a Maxwell–Stefan approach and mixture diffusion corrections. The balance contains a variable vapor density, ρ_p_, computed by the ideal gas law with local values p_p_, T_p_, and w_p,e_. The mass fraction of water in the gaseous permeate is w_p,w_ = 1 – w_p,e_, and the mole fractions are $x_{p,e}=\frac{w_{p,e}}{M_{e}}M_{p}$ and x_p,w_ = 1 – x_p,e_.

In the outflow (x = L), the normal diffusive flux is zero, i.e., the exiting flux is only convective. The lateral wall (r = R_f_ + L_p_) and the wall opposite to the outflow (x = 0) are insulated, and thus there are no mass fluxes of any component. The same type of permeable boundary as in the feed is set at r = R_f_, except that here, the total flux enters the permeate phase.

#### 2.1.4. Heat Transfer

*Feed side.* The stationary heat balance allows computing the temperature spatial distribution, T_f_, and it includes conductive and convective heat transfer rates.

(11) $$-\nabla \cdot \left(k_{f}\nabla T_{f}\right)+\rho_{f}C_{p,f}u_{f}\cdot \nabla T_{f}=0$$

where the thermal conductivity k_f_, the liquid density ρ_f_, and the heat capacity at constant pressure C_p,f_ depend on the local temperature and mixture composition (Table 1).

The liquid inflow (x = 0) has a set temperature T_f,in_, no conduction (i.e., no normal gradient of temperature) is assigned in the outflow (x = L), and axial symmetry (i.e., no heat flux) is set on the pipe axis at r = 0. On the membrane surface, water and ethanol evaporate and the liquid loses heat; therefore, an outward heat flux equal to the heat of vaporization is set at r = R_f_: $-k_{f}\nabla T_{f}=-q_{vap}$. The total vaporization heat flux is the sum of the two evaporating components, $q_{vap}=J_{w}L_{v,w}+J_{e}L_{v,e}$, computed using the latent heat of vaporization of water L_v,w_ and ethanol L_v,e_.

*Permeate side.* In the permeate (vapor phase), another heat balance is used to compute the spatial distribution of temperature T_p_ as follows:

(12) $$-\nabla \cdot \left(k_{p}\nabla T_{p}\right)+\rho_{p}C_{p,p}u_{p}\cdot \nabla T_{p}=0$$

where the thermal conductivity k_p_ and heat capacity C_p,p_ function as part of the local temperature and mixture composition, and density ρ_p_ is computed from the ideal gas law (Table 1).

The walls at x = 0 and r = R_f_ + L_p_ are insulated (adiabatic), whereas in the outflow (x = L), there is no more normal gradient of temperature (i.e., zero conduction). The membrane is assumed to be ideally conductive, so the temperature on the permeate side equals that on the feed side (T_p_ = T_f_ at r = R_f_).

#### 2.1.5. Parameters and Physical Properties

Model parameters include geometry dimensions and operating conditions (compositions, temperatures, pressures, and flow velocities). Furthermore, most physical properties are functions of temperature, and the mixture relations are also functions of composition (mole or mass fractions)—as presented in Table 1. Membrane permeability data and water–ethanol selectivity are also listed in Table 1.

**Table 1:** Model parameters, physical properties, and expressions for the average properties of the ethanol–water mixture.

Description	Symbol	Value or Expression					Units	Source and Comments
Module length	L	1000					mm	
Feed tube radius	R_f_	6					mm	
Permeate gap size	L_p_	2					mm	
Liquid feed inlet velocity	u_f,in_	2					cm/s	
Mass fractions in feed water ethanol	w_f,w,in_ w_f,e,in_	0.1 0.9					kg/kg	
Inlet temperature feed	T_f,in_	100					°C	373.15 K
Permeate outflow pressure	p_p,out_	3					kPa	Vacuum
Activity coefficients, liquid	$\ln\gamma_{i}$	$\frac{\sum_{j}x_{j}\cdot \tau_{ji}\cdot G_{ji}}{\sum_{k}x_{k}\cdot G_{ki}}+\sum_{j}\left(\frac{x_{j}\cdot G_{ij}}{\sum_{k}x_{k}\cdot G_{ki}}\cdot \left(\tau_{ij}-\frac{\sum_{m}x_{m}\cdot \tau_{mj}\cdot G_{mj}}{\sum_{k}x_{k}\cdot G_{kj}}\right)\right)$ $G_{ij}=\exp\left(-\alpha_{ij}\cdot \tau_{ij}\right)$ $\tau_{ij}=a_{ij}+\frac{b_{ij}}{T_{f}}$ $\alpha_{ij}=c_{ij}$ $c_{ij}=c_{ji}=\alpha$ $a_{ii}=a_{jj}=b_{ii}=b_{jj}=0$ a_ew_ = 0; a_we_ 0; b_ew_ = −55.1681; b_we_ = 670.441; c_ew_ = c_we_ = 0.3031;					- -	NRTL model equations [57,67,68] (T_f_ in K)
Vapor pressures	P_v,w_ P_v,e_	$\exp\left(65.93-\;7230/T_{f}-7.177\;\;\ln\;T_{f}+4.031\cdot 10^{-6}\;T_{f}^{2}\right)$ $\exp\left(86.49-\;7931/T_{f}-10.25\;\;\ln\;T_{f}+6.389\cdot 10^{-6}\;T_{f}^{2}\right)$					kPa kPa	[69] [69] (T_f_ in K)
Density, liquid mixture water ethanol	ρ_f_ ρ_w,L_ ρ_e,L_	$\frac{1}{w_{f,w}/\rho_{w,L}+w_{f,e}/\rho_{e,L}}$ $0.000010335\cdot T_{f}^{3}-0.013395\cdot T_{f}^{2}+4.969288\cdot T_{f}+432.2571$ $1038.799-0.850402\cdot T_{f}$					kg m^−3^ kg m^−3^ kg m^−3^	[69] [69] [69] (T_f_ in K)
Density, gas mixture	ρ_p_	$\frac{p_{p}M_{p}}{RT_{p}}$					kg m^−3^	Ideal gas law
Viscosity, liquid mixture water ethanol	η_f_ η_w,L_ η_e,L_	$\exp\left(x_{f,w}\;\ln\eta_{w,L}+x_{f,e}\;\ln\eta_{e,L}\right)$ $\begin{array}{l} 1.3799566-0.021224\cdot T_{f}+1.360456\cdot 10^{-4}\cdot T_{f}^{2}-4.645409\cdot 10^{-7}\cdot T_{f}^{3}\\ \;\;\;\;\;\;\;+8.904273\cdot 10^{-10}\cdot T_{f}^{4}-9.079069\cdot 10^{-13}\cdot T_{f}^{5}+3.845733\cdot 10^{-16}\cdot T_{f}^{6} \end{array}$ $\exp(1665.38568/T_{f}\;-\;12.399037)$					Pa s Pa s Pa s	[69] [69] [69] (T_f_ in K)
Viscosity, gas mixture water ethanol	η_p_ η_w,G_ η_e,G_	$x_{p,w}\;\eta_{w,G}+x_{p,e}\;\eta_{e,G}$ $\begin{array}{l} 3.798134\cdot 10^{-6}+2.778857\cdot 10^{-9}\cdot T_{p}+7.621016\cdot 10^{-11}\cdot T_{p}^{2}\\ -6.580738\cdot 10^{-14}\cdot T_{p}^{3}+2.07707\cdot 10^{-17}\cdot T_{p}^{4} \end{array}$ $\begin{array}{l} -9.85045\cdot 10^{-7}+3.361886\cdot 10^{-8}\cdot T_{p}-2.673253\cdot 10^{-12}\cdot T_{p}^{2}\\ -7.131648\cdot 10^{-15}T_{p}^{3}+3.44277\cdot 10^{-18}\cdot T_{p}^{4} \end{array}$					Pa s Pa s Pa s	[69] [69] [69] (T_f_ and T_p_ in K)
Thermal conductivity, liquid mixture water ethanol	k_f_ k_w,L_ k_e,L_	$x_{f,w}\;k_{w,L}+x_{f,e}\;k_{e,L}$ $-\;0.9003748+0.0083877\cdot T_{f}-1.118205\cdot 10^{-5}\cdot T_{f}^{2}$ $0.2571662-3.064779\cdot 10^{-4}\cdot T_{f}$					W m^−1^ K^−1^ W m^−1^ K^−1^ W m^−1^ K^−1^	[69] [69] [69] (T_f_ in K)
Thermal conductivity, gas mixture water ethanol	k_p_ k_w,G_ k_e,G_	$x_{p,w}\;k_{w,G}+x_{p,e}\;k_{e,G}$ $\begin{array}{l} 0.002953+2.573956\cdot 10^{-5}\cdot T_{p}+9.807821\cdot 10^{-8}\cdot T_{p}^{2}\\ -3.963389\cdot 10^{-11}\cdot T_{p}^{3}+1.026238\cdot 10^{-14}\cdot T_{p}^{4} \end{array}$ $k_{w,G}$					W m^−1^ K^−1^ W m^−1^ K^−1^ W m^−1^ K^−1^	[69] [69] (T_p_ in K) Assumed (negligible ethanol fraction in permeate)
Heat capacity const. P, liquid mixture water ethanol	C_p,f_ C_p,w,L_ C_p,e,L_	$w_{f,w}\;C_{p,w,L}+w_{f,e}\;C_{p,e,L}$ $4035.84079+0.492312034\cdot T_{f}$ $-384.391653+9.60563138\cdot T_{f}$					J kg^−1^ K^−1^ J kg^−1^ K^−1^ J kg^−1^ K^−1^	[69] [69] [69] (T_f_ in K)
Heat capacity const. P, gas mixture water ethanol	C_p,p_ C_p,w,G_ C_p,e,G_	$w_{p,w}\;C_{p,w,G}+w_{p,e}\;C_{p,e,G}$ $\begin{array}{l} 1745.96354+0.185114\cdot T_{p}+6.19448\cdot 10^{-4}\cdot T_{p}^{2}\\ \;\;\;\;\;\;\;\;\;\;\;\;\;-3.02678\cdot 10^{-7}\cdot T_{p}^{3}+4.19053\cdot 10^{-11}\cdot T_{p}^{4} \end{array}$ $\begin{array}{l} 878.1048-0.67455\cdot T_{p}+0.012544\cdot T_{p}^{2}\\ \;\;\;\;\;\;\;\;\;\;\;\;\;\;\;-1.59894\cdot 10^{-5}\cdot T_{p}^{3}+6.34290\cdot 10^{-9}\cdot T_{p}^{4} \end{array}$					J kg^−1^ K^−1^ J kg^−1^ K^−1^ J kg^−1^ K^−1^	[69] [69] [69] (T_p_ in K)
Molar mass mixture water ethanol	M_f_, M_p_ M_w_ M_e_	$\frac{1}{w_{f,w}/M_{w}+w_{f,e}/M_{e}}$, $\frac{1}{w_{p,w}/M_{w}+w_{p,e}/M_{e}}$ 18 46					g mol^−1^ g mol^−1^ g mol^−1^	
Latent heat of vaporization water ethanol		$\Delta H_{v}=C_{1}\cdot {\left(1-T_{r}\right)}^{\left(C_{2}+C_{3}\cdot T_{r}+C_{4}\cdot T_{r}^{2}\right)}$					J/kmol	[70] T_r_ = T/T_c_ (T_f_ in K)
		C_1_	C_2_	C_3_	C_4_	T_c_, K		
		5.66·10^7^	0.612041	−0.625697	0.398804	647.096		
		6.5831·10^7^	1.1905	−1.7666	1.0012	514		
Diffusion coefficient ethanol–water, liquid	D_f,we_	$1.3\cdot 10^{-9}\cdot 1.02^{T_{f}-298.15}$					m^2^ s^−1^	(T_f_ in K)
Diffusion coefficient ethanol–water, gas	D_p,we_	$2\cdot 10^{-5}$					m^2^ s^−1^	
Membrane permeances water ethanol	P_m,w_/L_m_ P_m,e_/L_m_	$3.54853\cdot 10^{-7}\cdot \exp(1.5771484\cdot a_{w})\cdot \exp[(E_{w}/R)\cdot (1/T_{ref}-1/T)]$ $1.07990\cdot 10^{-10}\cdot \exp(3.5393051\cdot a_{w})\cdot \exp[(E_{e}/R)\cdot (1/T_{ref}-1/T)]$ T_ref_ = 368.15 K E_w_ = 15,208.6 kJ/kmol E_e_ = 15,208.6 kJ/kmol					$\frac{\mathrm{kmol}}{m^{2}\cdot s\cdot \mathrm{kPa}}$ $\frac{\mathrm{kmol}}{m^{2}\cdot s\cdot \mathrm{kPa}}$	[57] [57]

#### 2.1.6. Model Solution

The numerical model was implemented and solved in the finite element modeling software COMSOL Multiphysics^®^ 6.3. The Chemical Reaction Engineering module of COMSOL was used to define all the model equations for the fluid flow (Single-Phase Laminar Flow), mass transfer (Transport of Concentrated Species), and heat transfer (Heat Transfer in Fluids). All partial differential equations used linear discretization schemes.

A mapped two-dimensional mesh of rectangular elements was used, with 100 equal elements over length x, 30 elements over feed tube radius R_f_ varying from 10 μm next to the membrane to 200 μm at the symmetry axis, and 20 elements over the permeate gap L_p_ with a size from 30 μm next to the membrane to 150 μm at the wall. 

For numerical stability, two stationary solver steps were used consecutively. The first is for isothermal flow and mass transfer, and the second also includes heat transfer, using the solution of the first step as the initial value.

### 2.2. Statistical Modeling and Optimization

To gain a deeper insight into how operational parameters and attributes influence the performance of PVA membranes, we conducted statistical modeling, generating virtual experiments in COMSOL. Considering a membrane surface of 2 m^2^ (to be relevant for practical applications), the efficiency of the pervaporation process was calculated based on the accurate model derived from fundamental principles and presented above.

The virtual experiments were carried out using a Box–Behnken design [14], according to the Response Surface Method (RSM) [15], considering three independent variables: feed water mass fraction, feed temperature, and permeate pressure.

The dependent variables calculated from the simulation results were the relative ethanol enrichment in the retentate (Equation (13)) and rejection percentages of the membranes (Equation (14)).

(13) $$RE\;(\%)=\frac{w_{R,e}-w_{F,e}}{w_{F,e}}\cdot 100$$

(14) $$s\;(\%)=\frac{R\cdot w_{R,e}}{F\cdot w_{F,e}}\cdot 100$$

where w_R,e_ and w_F,e_ are ethanol mass fractions in retentate and feed, respectively, and R and F are retentate and feed mass rates.

Table 2 outlines the range of parameter variations in the virtual experimental study, using the values from the COMSOL model as a central point (Table 1).

The details of the experiments conducted as a part of this investigation are shown in Table 3 and are presented in the Section 3.

The regression models for RE and s were further considered as criteria for multiobjective optimization, aiming for their simultaneous maximization, which was performed using the global optimization tools implemented in MATLAB R2025.

## 3. Results and Discussion

### 3.1. Pervaporation Theoretical Model in COMSOL Multiphysics^®^

**Figure 2:**
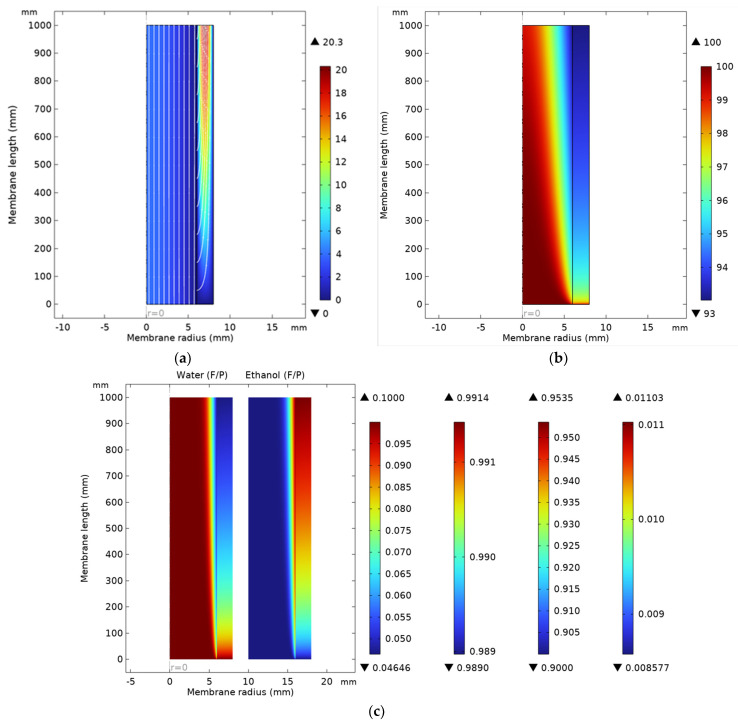
Profiles in the feed and permeate compartments: (**a**) velocities (cm/s); (**b**) temperature (°C); (**c**) water and ethanol mass fraction (–).

Based on the model considering the flow, mass, and heat transfer equations implemented in COMSOL, the velocities, temperatures, and concentration profiles were obtained, and they are presented in Figure 2. The surface-type graphs show the axisymmetric 2D section through the membrane module.

Figure 2a illustrates the laminar profile of the flow (maximum velocity values in the center of each compartment). Higher velocities are noticed in the permeate compartment, as the permeate is in the vapor phase. As one can see in Figure 2b, the temperature in the retentate (feed compartment) decreases due to the vaporization process during pervaporation through the membrane, and as expected, the permeate is cooler than the retentate.

Figure 2c presents both water and ethanol profiles in the same 2D section of the membrane. The origin of the ethanol mass fraction representation is translated, so the symmetry axis appears at the value of 10 mm. The first two color bars refer to the water concentrations in feed and permeate, respectively, while the ethanol concentration legends are given by the other two color bars. It has to be noticed that the color scale is different, as it is associated with different concentration ranges for water and ethanol, respectively.

The water concentration decreases along the membrane in the feed due to the permeation process and also in the permeate due to simultaneous mass transfer of the ethanol through the membrane, even if this is less important. On a 1 m long membrane, the decrease in water concentration in the feed compartment is about 5%, while in the permeate, it is only ~0.2%. The ethanol mass fraction profile proves its concentration in the retentate (feed compartment) from 0.9 to 0.9535.

**Figure 3:**
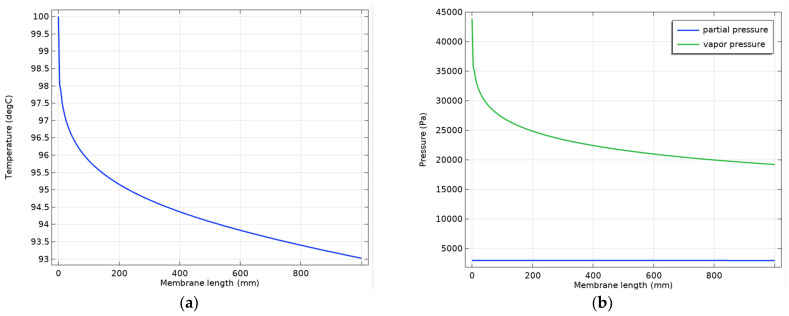
Variations along the membrane: (**a**) temperature; (**b**) driving force for water permeation.

As the PVA membrane is selective for water, the process driving force of the permeation is the difference between the water vapor pressure and the water partial pressure in the permeate $\left(\gamma_{w}x_{f,w}P_{v,w}-x_{p,w}p_{p}\right)$. Figure 3 presents the variation of the temperature and this driving force along the membrane.

As one can see in Figure 3a, the temperature in the feed compartment decreases along the membrane due to the vaporization of the permeate. For the 1 m long membrane, the decrease is maybe not important (only 7 degrees), but it leads to a reduction in driving force (Figure 3b).

Analyzing the modeling results, one can say that the COMSOL model proves to be consistent with the theoretical description of the pervaporation process, and the studied PVA membrane shows a good capacity to concentrate the ethanol in solutions near azeotropic mixtures.

Moreover, the values of water fluxes through the membrane calculated in COMSOL were compared (Table 3) with the literature reports [52,57] for the same operating conditions.

To extend the study to more practical applications, we used this model to simulate the PVA membrane performance on a larger scale (a membrane surface of about 2 m^2^).

### 3.2. Statistical Modeling and Optimization

The virtual experimental values for relative enrichment of ethanol in retentate RE (%) and the ethanol recuperation degree s (%) for every combination of the operating parameters, according to the Box–Behnken program, are presented in Table 4.

The second-degree models were proposed in terms of coded variables for both the relative enrichment of ethanol in retentate and the ethanol recuperation degree. The general relation of these models is as follows:

(15) $$y=b_{0}+b_{1}\cdot x_{1}+b_{2}\cdot x_{2}+b_{3}\cdot x_{3}+b_{12}\cdot x_{1}\cdot x_{2}+b_{13}\cdot x_{1}\cdot x_{3}+b_{23}\cdot x_{2}\cdot x_{3}+b_{11}\cdot x_{1}^{2}+b_{22}\cdot x_{2}^{2}+b_{33}\cdot x_{3}^{2}$$

where y stands for the relative enrichment of ethanol in retentate RE and the ethanol recuperation degree s, respectively.

#### 3.2.1. Model for Relative Enrichment of Ethanol in Retentate

The model coefficients were calculated by regression, and the values and their significance are presented in Table 5. According to Fisher’s test, the model is significant at a level of 5% (*p*-value = 1.453·10^−4^). The determination coefficient R^2^ for the model is 0.9993, and the adjusted R^2^ is slightly lower, 0.9972.

The minor influence of x_3_^2^ and the interactions between permeate pressure and feed concentration (x_1_·x_3_) and permeate pressure and temperature (x_2_·x_3_) is confirmed by the analysis of the significance of the model coefficients—the *p*-value (Table 5).

(16) $$\begin{array}{ll} RE\;(\%) & =4.14222+1.557732\cdot x_{1}+1.626906\cdot x_{2}-0.249572\cdot x_{3}+0.568607\cdot x_{1}\cdot x_{2}-0.019582\cdot x_{1}\cdot x_{3}+\\ & +0.045\cdot x_{2}\cdot x_{3}-0.26554\cdot x_{1}^{2}+0.04834\cdot x_{2}^{2}+0.001664\cdot x_{3}^{2} \end{array}$$

**Figure 4:**
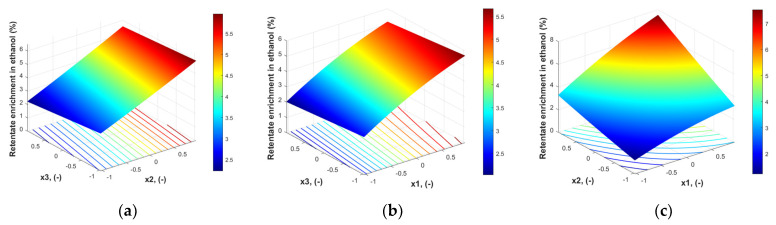
Variation of retentate enrichment in ethanol (%) with the operating condition expressed in coded values: (**a**) x_1_ = 0, (**b**) x_2_ = 0, (**c**) x_3_ = 0.

The response surface corresponding to this model (Equation (16)) is given in Figure 4.

As Figure 4 shows, the response surface does not exhibit a clear maximum. Some high retentate enrichment in ethanol values can be reached in a region characterized by higher values of x_1_ and x_2_ and lower values of x_3_.

#### 3.2.2. Model for Ethanol Recuperation Degree

The general model proposed for ethanol recuperation degree is given by Equation (17), and the coefficients of the full second-order degree model obtained are given in Table 6. The coefficient of determination is R^2^ = 0.9959, the adjusted R^2^ is 0.9837, and the Fisher test confirms the significance of the model (*p*-value = 0.001998).

The model used in this study is as follows:

(17) $$\begin{array}{ll} s\;(\%) & =99.303-0.28225\cdot x_{1}-0.0395\cdot x_{2}+0.07575\cdot x_{3}+0.00425\cdot x_{1}\cdot x_{2}+0.03625\cdot x_{1}\cdot x_{3}-\\ & -0.02475\cdot x_{2}\cdot x_{3}+0.002375\cdot x_{1}^{2}+0.040875\cdot x_{2}^{2}+0.002375\cdot x_{3}^{2} \end{array}$$

**Figure 5:**
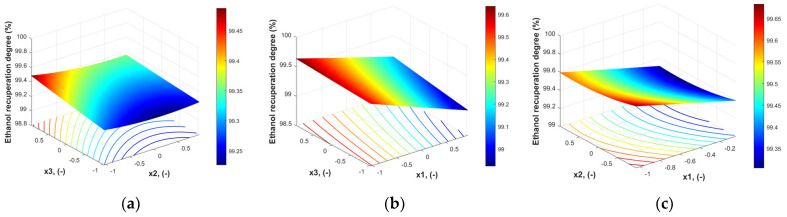
Ethanol recuperation degree (%) with operating condition expressed in coded values: (**a**) x_1_ = 0, (**b**) x_2_ = 0, (**c**) x_3_ = 0.

A more relevant image of the response surface is given in Figure 5.

As Figure 5 shows, the response surface for ethanol recuperation degree does not exhibit a clear maximum. Some high recuperation degrees of ethanol can be reached at lower values of x_1_ and x_2_ and higher values of x_3_, which is a contradictory trend compared to the retentate enrichment in ethanol.

This dichotomy leads to the need to find ways to optimize both objectives. The objective functions considered for the optimization are expressed by the polynomial models obtained (Equations (16) and (17)).

These two objective functions must be maximized simultaneously in order to identify operating conditions that improve membrane performance. Traditional optimization methods attempt to combine conflicting objectives either by assigning weights and merging them into a single function or by defining a desirability index for each criterion and aggregating these into an overall desirability function. Both approaches require predefined scaling and restriction of the solution space, ultimately yielding a single optimal point [71].

In this study, genetic algorithms (GAs) were selected to address the multiobjective optimization problem. Because they evaluate many candidate solutions in parallel, GAs are particularly well-suited for identifying Pareto fronts rather than single optimal solutions. Unlike scalarization techniques that require multiple runs to explore various trade-offs, genetic algorithms generate a diverse set of Pareto-optimal solutions within a single evolutionary process.

**Figure 6:**
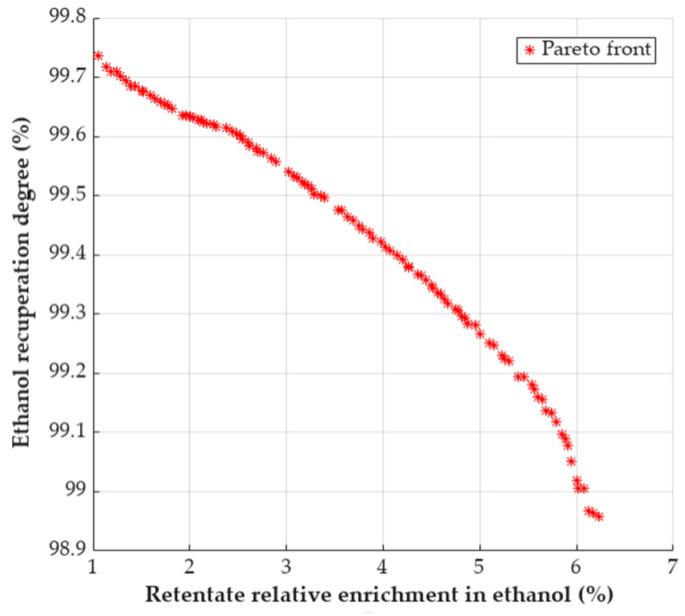
Pareto front for simultaneous maximization of ethanol recuperation degree and retentate relative enrichment in ethanol.

The results obtained are presented in Figure 6, where the Pareto front is given.

The set of equally good solutions that represent the Pareto front shows that there are some combinations of working conditions that can ensure a trade-off between the two criteria considered.

Some selected sets from the Pareto front can also provide more insight into how operating parameters influence the PVA membrane efficiency (Table 7).

In case of mixtures with lower water content, the operating conditions can be milder in terms of temperature and permeate pressure (~80 °C, 5 kPa), obtaining a very high ethanol recuperation degree. If the temperature is raised to about 100 °C, better retentate enrichment in ethanol can be achieved, without significantly decreasing the recuperation degree.

It is worth noticing that similar operating conditions are suitable for mixtures with higher water content, realizing a significant increase in ethanol concentration. Higher enrichment can be achieved at the cost of decreasing permeate pressure and reducing ethanol recovery.

## 4. Conclusions

The performance of a PVA membrane applied in pervaporation was investigated through COMSOL-based modeling and simulation, targeting larger-scale ethanol dehydration. In this first modeling attempt, which integrates flow, mass, and heat transfer for a PVA membrane with well-established separation properties, the simulations demonstrated strong predictive capability, delivering results consistent with previously reported experimental data and calculated performance indicators.

Using virtual experiments designed according to a Box–Behnken scheme within the RSM framework, the variation of separation performance was mapped across a large range of operating conditions. The statistical modeling yielded original analytical expressions for two key criteria—ethanol recovery degree and relative retentate enrichment—as functions of the main process variables.

A multiobjective optimization effort, focusing on the simultaneous maximization of these two criteria, was carried out using a genetic algorithm implemented in MATLAB, searching on a practically relevant range of operating parameters. This approach revealed a Pareto front of equally optimal solutions from which meaningful trade-offs between ethanol recovery (s) and retentate relative enrichment (RE) were identified.

For example, the Pareto front offered some combination of temperature and pressure that can lead to good trade-offs between separation degree (s) and relative enrichment of retentate in ethanol (RE) for concentrated ethanol solutions (around 0.05 water mass fraction). For more diluted ethanol solutions (around 0.15 water mass fraction), which are more easily obtained by distillation, some combinations of operating parameters are also available (Table 7). Since pervaporation units are often supplied with the top product from a distillation column, a more diluted distillate is associated with potential energy savings.

The combined methodology—COMSOL modeling based on coupled flow, heat, and mass transfer equations, RSM-based statistical exploration, and multiobjective optimization—proved to be an effective and original approach for evaluating the capability of PVA membranes in ethanol dehydration. Since PVA-based membranes are already used in practical applications, this integrated strategy can be further extended to industrial-scale analyses.

## Figures and Tables

**Table 2:** The different levels of experimental variables.

Variables (Operating Parameters)	Coded Variables	Coded Levels and Actual Values		
		−1	0	1
Water content in feed, w_w_in_ (mass fraction)	x_1_	0.05	0.1	0.15
Feed temperature, T_f_in_ (°C)	x_2_	80	100	120
Permeate pressure, p_p_ (kPa)	x_3_	1	3	5

**Table 3:** Experimental and calculated values.

Temperature (°C)	Water Flux $\left(\frac{\mathrm{kg}}{m^{2}\cdot h}\right)$	Water Partial Pressure (kPa)	Water Activity (−)	Water Permeance $\left(\frac{\mathrm{kmol}}{m^{2}\cdot s\cdot \mathrm{kPa}}\right)$	Obs.
95	1.6 ÷ 1.7 *				Measured by Yave [52]
	1.6406				This work
60	0.202	9.0425	0.45364	3.8719·10^−7^	Estimated by Vane [57] using experimental data from Yave [52]
	0.223	8.99	0.45350	4.2943·10^−7^	This work
105	2.400	52.346	0.43316	7.2070·10^−7^	Estimated by Vane [57] using experimental data from Yave [52]
	2.650	52.055	0.4329	7.982·10^−7^	This work

* Reading from a graph.

**Table 4:** Virtual experimental data Box–Behnken design.

Run #	x1	x2	x3	Relative Enrichment of Ethanol in Retentate (%)	Ethanol Recuperation Degree (%)
1	1	1	0	7.6588	99.029
2	1	−1	0	3.3718	99.126
3	−1	1	0	3.3411	99.558
4	−1	−1	0	1.3284	99.672
5	1	0	1	5.1365	99.122
6	1	0	−1	5.6706	98.902
7	−1	0	1	2.1253	99.641
8	−1	0	−1	2.5811	99.566
9	0	1	1	5.6644	99.373
10	0	1	−1	6.0778	99.267
11	0	−1	1	2.2167	99.475
12	0	−1	−1	2.8100	99.270
13	0	0	0	4.1422	99.303

**Table 5:** ANOVA results for the relative enrichment of ethanol in the retentate model.

	Coefficients	Standard Error	*p*-Value
Intercept	4.14222	0.10021	3.1161·10^−5^
x_1_	1.55773	0.03543	2.59·10^−5^
x_2_	1.62691	0.03543	2.2739·10^−5^
x_3_	−0.24957	0.03543	0.00588
x_1_·x_2_	0.56861	0.05011	0.00147
x_1_·x_3_	−0.01958	0.05011	0.72204
x_2_·x_3_	0.04500	0.05011	0.43532
x_1_^2^	−0.26554	0.06628	0.02790
x_2_^2^	0.04834	0.06628	0.51864
x_3_^2^	0.00166	0.06628	0.98155

**Table 6:** ANOVA results for the ethanol recuperation degree model.

	Coefficients	Standard Error	*p*-Value
Intercept	99.30300	0.03106	6.74935·10^−5^
x_1_	−0.28225	0.01098	0.00013
x_2_	−0.03950	0.01098	0.03685
x_3_	0.07575	0.01098	0.00624
x_1_·x_2_	0.00425	0.01553	0.80211
x_1_·x_3_	0.03625	0.01553	0.10177
x_2_·x_3_	−0.02475	0.01553	0.20928
x_1_^2^	0.00238	0.02055	0.91528
x_2_^2^	0.04088	0.02055	0.14075
x_3_^2^	0.00238	0.02055	0.91528

**Table 7:** Parameter values selected from the Pareto front in multiobjective optimization, considering maximum ethanol recuperation degree and maximum retentate relative enrichment in ethanol.

	Variables						Objective Functions	
	Coded Values			Real Values			RE (%)	s (%)
	x_1_	x_2_	x_3_	Water Content in Feed, w_F,w_ (Mass Fraction)	Feed Temperature, T_f,in_ (°C)	Permeat Pressure, p_p_ (kPa)		
Diluted solutions	0.9429	0.2907	0.3295	0.1471	105.81	3.66	5.9241	99.0662
	0.9529	0.2951	0.9639	0.1476	105.90	4.93	5.7855	99.1308
	1.0000	0.3000	−0.9764	0.1500	106.00	1.05	6.3486	98.9164
Concentrated solutions	−0.9918	0.2282	0.9779	0.0504	104.56	4.96	2.3677	99.6131
	−0.9841	−0.0439	0.9619	0.0508	99.12	4.92	2.0835	99.6268
	−0.9826	−0.9680	0.9943	0.0509	80.64	4.99	1.0958	99.7293

## Data Availability

The original contributions presented in this study are included in the article. Further inquiries can be directed to the corresponding author.
